# How do different perceived school goal structures affect Chinese kindergarten teachers’ professional identity? The role of basic psychological needs satisfaction and growth mindset

**DOI:** 10.3389/fpsyg.2025.1588334

**Published:** 2025-04-29

**Authors:** Wei Luo, Xingcan Ni, Chundie Wang, Jiaqin Wang, Yujia Zeng, Chengfu Yu

**Affiliations:** ^1^School of Education, Guangdong Polytechnic, Foshan, China; ^2^Department of Psychology/Research Center of Adolescent Psychology and Behavior, School of Education, Guangzhou University, Guangzhou, China

**Keywords:** perceived school goal structure, professional identity, basic psychological needs satisfaction, growth mindset, kindergarten teacher

## Abstract

**Introduction:**

Due to the unique challenges and high-intensity emotional labor in their work, kindergarten teachers’ high levels of job burnout and turnover rate have become a widely recognized social issue. To address this situation, exploring the key factors that enhance kindergarten teachers’ professional identity is of paramount importance. Professional identity development is influenced not only by the cultural contexts and workplace environment but also by individual teacher characteristics. However, existing research on how environmental and psychological factors interact to influence the professional identity of kindergarten teachers is scarce.

**Methods:**

This study examined the effects of perceived school goal structure on kindergarten teachers’ professional identity and explored the mediating role of basic psychological needs satisfaction (BPNS) and the moderating role of growth mindsets. A questionnaire survey was conducted with 1,475 kindergarten teachers from China, selected using random sampling.

**Results:**

The results demonstrated that school learning and performance goal structures indirectly affected teachers’ professional identity through the mediating role of BPNS; however, only the school learning goal structure directly affected professional identity. Additionally, growth mindsets only moderated the relationship between school performance goal structure and BPNS. Regardless of the school performance goal structure, teachers with strong growth mindsets reported higher levels of BPNS than those with weak growth mindsets.

**Discussion:**

This study provides a solid theoretical foundation and practical guidance for improving teachers’ professional identity within the unique sociocultural context of China.

## 1 Introduction

Teachers exert a profound influence on societal progress. To ensure the wellbeing of both individuals and society as a whole, it is imperative to recognize the significance of teachers and support their professional development (Wu et al., 2024). Teachers’ professional identity has long been regarded as the psychological foundation of their professional development (Hanna et al., 2020). Professional identity is defined as an individual’s recognition and understanding of the beliefs, values, attitudes, and roles associated with their professional group (Abreu, 2006), involving an individual’s cognitive understanding of their professional role, emotional connection, and behavioral manifestations (Zhang et al., 2016). Teachers with a strong professional identity often exhibit a high degree of intrinsic motivation, viewing teaching as a lasting and passionate pursuit rather than merely a means of livelihood (Wu et al., 2024).

Teachers with high professional identity experience high wellbeing (Hui et al., 2024) and job satisfaction (Wu et al., 2024). Conversely, low professional identity leads to a high probability of teachers leaving their jobs (Wang et al., 2024), which, in turn, affects teaching quality (Li, 2021). Kindergarten teachers are the first resource for the high-quality development of preschool education. As high-intensity emotional laborers (Yuan et al., 2025), kindergarten teachers are often prone to emotional exhaustion, job burnout, and even high turnover intentions (Wang et al., 2024). Professional identity can encourage teachers to grow by allowing them to actively perceive and evaluate career-related things (Li, 2014), and it is a particularly crucial aspect in improving emotional fatigue and high turnover rates among early childhood educators. Therefore, exploring the determinants affecting the professional identity of kindergarten teachers is critical for enhancing their psychological wellbeing and improving the quality of education.

The development of professional identity is influenced by cultural context and work environment as well as individual teacher characteristics. However, existing research on how environmental and psychological factors interact to shape the professional identity of kindergarten teachers is scant. Teachers are renowned for their intrinsic motivation and dedication to their profession (Wu et al., 2024); hence, we drew on two complementary motivational theories (Boncquet et al., 2024), namely, Dweck’s social-cognitive framework (Dweck and Leggett, 1988) and the self-determination theory (Deci and Ryan, 1985). We examined the relationship between different school goal structures and teacher professional identity and tested the role of basic psychological need satisfaction (BPNS) and growth mindset in this relationship. By jointly examining the two theories, we aimed to uncover the influencing mechanism of teacher professional identity from two different motivation levels.

### 1.1 Perceived school goal structure and professional identity

Teachers’ professional identity is a socialization result of individual cognition (Le Maistre and Paré, 2010) influenced by environmental and sociocultural factors (Gibbons, 2020). Teachers in different cultural contexts and school environments may experience varying levels of professional identity. Perceived school goal structure is the application of achievement goal orientation in understanding school goal orientation. It refers to teachers’ interpretations and internalization of the goals and values promoted by their educational institutions, and can be divided into school learning and performance goal structures (Zhang, 2010). School learning goal structure emphasize acquiring knowledge and skills through persistence, personal learning, and mastering new tasks and view mistakes as part of the learning process. By contrast, school performance goal structure focus on performing well, achieving success through competition, and outperforming others (Skaalvik and Skaalvik, 2011).

According to Dweck’s social-cognitive framework (Dweck and Leggett, 1988), achievement goals direct individuals’ attentional focus and influence their evaluation of events, fostering distinct achievement-related emotions. Similarly, perceived school goal structures can promote the development of different achievement emotions in individuals. When schools encourage teachers to engage in professional development and self-improvement, they are more likely to be motivated by intrinsic factors (i.e., their interest in and satisfaction with the teaching profession), strengthening their professional identity (Xie et al., 2018). Conversely, school performance goal structure emphasizing achievement and competition may lead teachers to focus on external rewards and avoid failure. Although this type of goal structure may improve short-term performance, it can also suppress teachers’ autonomy and intrinsic motivation (Luo et al., 2020), thus harming their professional identity (Xie et al., 2018).

Several studies have indirectly highlighted the importance of school goal structures in influencing teachers’ professional identity. For instance, Skaalvik and Skaalvik (2023) showed that both types of perceived school goal structures can directly influence teachers’ self-efficacy, and perceived performance goal structure negatively impact teachers’ engagement. Sun et al. (2025) analyzed three types of teacher burnout patterns. They found that teachers with a performance goal structure are more likely to belong to the achievement loss and emotional exhaustion groups, while teachers with a mastery class goal structure are less likely to belong to these groups. This result suggests that the goal structure type perceived by teachers can significantly affect their teaching behaviors and emotions. However, how different perceived school goal structures influence kindergarten teachers’ professional identity within the unique sociocultural context of China is not well understood.

### 1.2 Mediating role of basic psychological needs satisfaction

Self-determination theory states that BPNS is the basis for an individual’s intrinsic motivation (Deci and Ryan, 1985). Individuals show high intrinsic motivation, positive mental health, and strong behavioral adaptability only when the external environment satisfies their basic psychological needs for autonomy, belonging, and competence. In other words, BPNS may be a key intrinsic mechanism in connecting perceived school goal structure and kindergarten teachers’ professional identity.

Environmental factors can either facilitate or impede a teacher’s BPNS (Cai and Tang, 2022). The school goal structure is a crucial environmental factor influencing teachers’ BPNS. Schools that adopt a learning goal structure encourage teachers and students to focus on mastering the teaching activities, which are controllable, valuable, and consistent with the teachers’ educational philosophies. This focus promotes positive experiences for teachers during the teaching process (Luo et al., 2020). In such an environment, teachers can experience a degree of autonomy and agency and make decisions that align with their values and beliefs, satisfying their basic psychological needs (Ryan and Deci, 2020). In contrast, school performance goal structure places greater emphasis on external competition and comparison rather than on individual growth, which can undermine teachers’ BPNS, such as autonomy and belonging (Ketonen and Nieminen, 2025; Nerstad et al., 2020; Schwarz-Franco and Hadar, 2024). Moreover, BPNS affects teachers’ professional identity. When teachers’ basic psychological needs are met, they are more likely to perceive their professional abilities as being recognized (Xu et al., 2023), thereby developing a healthy sense of self and a stronger professional identity (Weiß et al., 2023). A previous study reported that pre-service teachers who felt strong autonomy support, a high sense of competence, and positive associations with others had strong professional identity (Wong and Liu, 2024).

Some studies have indirectly demonstrated the role of BPNS. For instance, Liu et al. (2024) found that BPNS can mediate the impact of parent-initiated support on career calling among Chinese kindergarten teachers. Wu et al. (2023) found that BPNS and autonomous motivation acted as a mediator between school principals’ need-supporting styles and teachers’ approaches to job crafting. Similarly, Lee et al. (2020) showed that BPNS significantly mediated perceived principal’s learning support and teachers’ change-oriented work behaviors. These studies suggest that BPNS can mediate the relationship between the supportive styles of the work environment and teachers’ positive teaching styles. However, research exploring how BPNS mediates the link between kindergarten teachers’ perceived school goal structure and professional identity is limited.

### 1.3 Growth mindset as a moderator

Mindsets influence the cognitive processing of information, leading individuals to perceive things differently (Chen and Usher, 2013). Therefore, individuals with different mindsets may experience varying degrees of BPNS when facing the same school goal structure. Dweck’s social-cognitive framework distinguishes between fixed and growth mindsets (Dweck and Leggett, 1988). A growth mindset is a positive way of considering the malleability of personal qualities (Dweck and Leggett, 1988). Unlike a fixed mindset that perceives intelligence and ability as unchangeable, a growth mindset believes these qualities can be enhanced or developed through continuous learning and effort (Dweck and Leggett, 1988). Similar to the impact of a school learning goal structure, when adopting a growth mindset, teachers are more likely to recognize the value of their own and their students’ progress (Bardach et al., 2024). This phenomenon induces a positive motivational pattern among teachers (Dweck and Leggett, 1988), as it directs them toward learning and improvement and positively influences their self-efficacy (Bardach et al., 2024), life satisfaction (Lee et al., 2023), and wellbeing (Nalipay et al., 2022).

According to the job demands-resources model (Bakker and Demerouti, 2007), work imposes various demands on individuals, including physical, psychological, social, and organizational aspects. These demands can lead to fatigue and tension; however, job resources help individuals cope effectively, promoting engagement and wellbeing. A growth mindset is a meaningful psychological resource that helps teachers cope with job demands and improves their wellbeing (Zilka et al., 2023). Therefore, BPNS, a factor of wellbeing (Keller et al., 2024), may be positively influenced by growth mindsets in the process of teachers handling job demands, such as the requirements of school goal structure. In other words, growth mindsets may interact with perceived school goal structure to positively affect BPNS among kindergarten teachers. Teachers with growth mindsets may adapt to perceived school goal structure, adjust their teaching strategies and methods, and perceive the requirements and challenges as opportunities for growth and progress rather than stresses and burdens. These teachers continuously challenge and surpass themselves in achieving school goals, attaining a high sense of achievement and satisfaction (Lee et al., 2023).

Although previous studies have not examined growth mindsets as a moderating variable in the analysis of BPNS among kindergarten teachers, some empirical studies have provided indirect support. For instance, Jiang et al. (2024) found that growth mindsets positively moderated the positive impact of two external environmental aspects— institutional integrity and perceived teacher support— on the thriving of students in private higher education institutions. Brandisauskiene et al. (2021) revealed that students’ growth mindsets moderated the positive connection between teacher support and student accomplishment. This positive connection was significant only among students with strong growth mindsets. Moreover, a study demonstrated that perceived school climate interacted with growth mindsets to jointly enhance teachers’ work engagement in China (Zhang and He, 2024). These studies indicate that growth mindsets play a significant role in strengthening the positive impact of school environmental factors on individual development.

### 1.4 The present study

**Figure 1:**
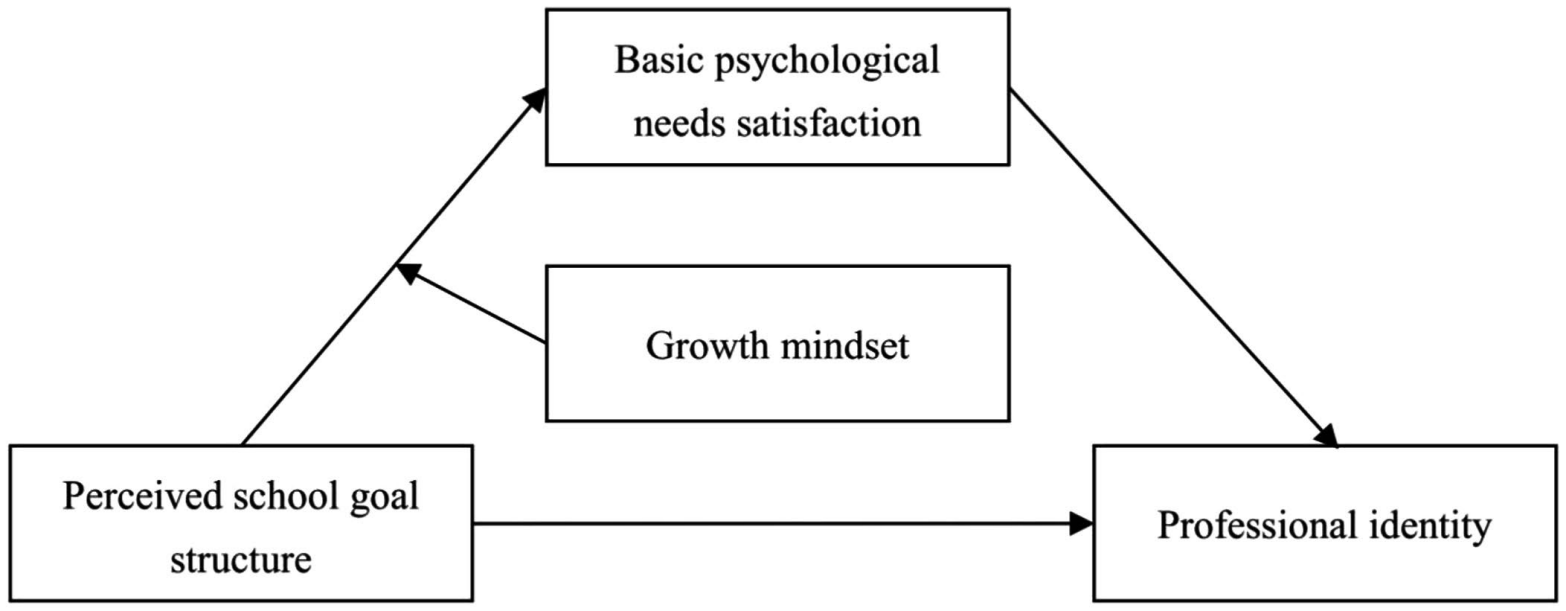
Hypothesis model.

To fill the gaps in the existing literature, this study examined the effects of perceived school goal structures on professional identity among Chinese kindergarten teachers. It explored the mediating role of BPNS and the moderating role of growth mindsets in this relationship (Figure 1). Based on previous research findings, we proposed the following hypotheses:

*H1*: School learning goal structure is positively associated with kindergarten teachers’ professional identity, whereas school performance goal structure is negatively associated with their professional identity.

*H2*: BPNS significantly mediates the relationship between perceived school goal structures and professional identity.

*H3*: Growth mindsets significantly moderate the relationship between perceived school goal structure and BPNS.

## 2 Methods

### 2.1 Participants

This study randomly selected multiple kindergartens across the country willing to participate in the research and invited all teachers from these kindergartens to complete a questionnaire survey. A total of 1,627 teachers participated in the survey. Subsequently, 152 questionnaires were excluded due to incomplete answers, completion times of less than 180 s and abnormal age filling. Finally, 1,475 kindergarten teachers (1,432 women and 43 men) were included in the analysis. The participants’ ages ranged from 20 to 60 years, with an average of 34.01 years (*SD* = 8.41 years). Their work experience ranged from 0 to 43 years (*M* = 10.92 years, *SD* = 9.33 years).

### 2.2 Procedure

For convenience, data were collected using the Wenjuanxing platform^1^. Ethical clearance for this research was obtained from the Academic Ethics Review Board at the researchers’ university. After obtaining informed consent, we distributed the questionnaire link to each teacher. All respondents were required to fill in basic identifiable information and questionnaires on perceived school goal structure, professional identity, BPNS, and growth mindsets.

### 2.3 Measures

#### 2.3.1 Perceived school goal structure

A two-dimensional goal structure scale was used to gauge teachers’ perceived school goal structure (Skaalvik and Skaalvik, 2011). Prior research has demonstrated that the scale is sufficiently valid and reliable (Skaalvik and Skaalvik, 2013). This scale includes six items in two dimensions: school learning goal structure (e.g., “My school places great emphasis on creating a safe and inspiring learning environment”) and school performance goal structure (e.g., “The school I teach emphasizes students’ scores in academic tests very much”). Responses were rated on a six-point scale (1 = *completely disagree*; 6 = *completely agree*). A higher average score indicates a greater degree of perceived school goal structure. The Cronbach’s *α* of the perceived school learning and performance goal structure scales in this study were 0.93 and 0.94, respectively.

#### 2.3.2 Basic psychological needs satisfaction

BPNS was measured using the satisfaction subscale of the Basic Psychological Need Scale (Deci and Ryan, 2000). The relevant measure has demonstrated adequate reliability and validity in Chinese kindergarten teachers (Song et al., 2021). This scale comprises 12 items (e.g., “People who often deal with me tend to consider my feelings”). Answers were rated on a seven-point scale (1 = *completely disagree*; 7 = *completely agree*). A higher average score indicates greater BPNS. The Cronbach’s *α* was 0.97.

#### 2.3.3 Growth mindset

Growth mindset was measured using the Growth Mindset Scale (Dweck, 2006). This scale has shown adequate reliability and validity in studies addressing Chinese teachers (Liu et al., 2025). The scale comprises three growth mindset items and three fixed mindset items (e.g., “I can learn new knowledge, but my intelligence level cannot be changed”). Responses were rated on a seven-point scale (1 = *completely disagree*; 7 = *completely agree*). The fixed mindset items were reverse-scored. A higher average score indicates a greater likelihood of having a growth mindset. The Cronbach’s α was 0.60.

#### 2.3.4 Professional identity

Professional identity was measured using the Teacher Professional Identity Scale’s professional belonging and professional efficacy subscale (Li and Yan, 2018). Previous studies have shown good reliability and validity for this scale among Chinese teachers (Wang, 2025). The scale comprises nine items (e.g., “Being a teacher can realize my life value”). Responses were rated on a five-point scale (1 = *completely disagree*; 5 = *completely agree*). A higher average score indicates a higher degree of professional identity. The Cronbach’s α was 0.95.

#### 2.3.5 Teaching performance

The teaching performance was measured using a single item: “Compared to others, how would you rate your teaching performance?” Responses were rated on a five-point scale (1 = *very bad*; 5 = *very good*). A higher score indicates a higher degree of teaching performance.

### 2.4 Data analysis

IBM SPSS 27.0 was used for all statistical analyses. Descriptive statistics and correlation analyses were conducted for all variables. The mediating role of BPNS and the moderating effect of a growth mindset were analyzed using SPSS PROCESS 4.0 and Macro Models 4 and 7 (Hayes, 2015). We assessed the unconditional indirect effects using a bootstrapping approach that comprised 5,000 resamples. The impact was considered statistically significant when the 95% confidence interval (CI) did not include zero. All continuous variables were standardized. Both models controlled for gender, age, teaching performance, and socioeconomic status. When examining the model of school learning goal structure on professional identity, the school performance goal structure was controlled; when reviewing the model of school performance goal structure on professional identity, the school learning goal structure was controlled.

## 3 Results

### 3.1 Descriptive and correlational analyses

Descriptive statistics and correlation analysis revealed that school learning goal structure was significantly and positively correlated with school performance goal structure, BPNS, growth mindset, and professional identity (Table 1). School performance goal structure was positively correlated with BPNS and professional identity. BPNS was positively correlated with a growth mindset and professional identity. Moreover, a growth mindset was significant and positively correlated with professional identity.

**Table 1:** Correlations and descriptive statistics of the variables.

Variable	1	2	3	4	5	6	7	8	9
1. Gender	1.00								
2. Age	0.04	1.00							
3. TP	0.05	0.23***	1.00						
4. SES	0.04	−0.12***	0.07**	1.00					
5. SLGS	−0.05*	0.08**	0.20***	0.07**	1.00				
6. SPGS	0.03	−0.08**	0.08**	0.00	0.25***	1.00			
7. BPNS	0.02	0.15***	0.22***	0.11***	0.48***	0.23***	1.00		
8. GM	−0.02	−0.04	0.08**	0.10***	0.13***	−0.02	0.18***	1.00	
9. PI	0.02	0.17***	0.25***	0.08**	0.49***	0.17***	0.74***	0.09***	1.00
*M*	−	34.01	3.74	2.80	5.61	4.60	6.02	3.70	4.43
*SD*	−	8.41	0.83	0.77	0.65	1.56	1.01	0.82	0.64

*p < 0.05, **p < 0.01, ***p < 0.001. Gender was coded as 0 = man and 1 = woman. TP = teaching performance; SES = socioeconomic status; SLGS = school learning goal structure; SPGS = school performance goal structure; BPNS = basic psychological needs satisfaction; GM = growth mindset; PI = professional identity.

### 3.2 Tests of mediating effects of basic psychological needs satisfaction

**Figure 2:**
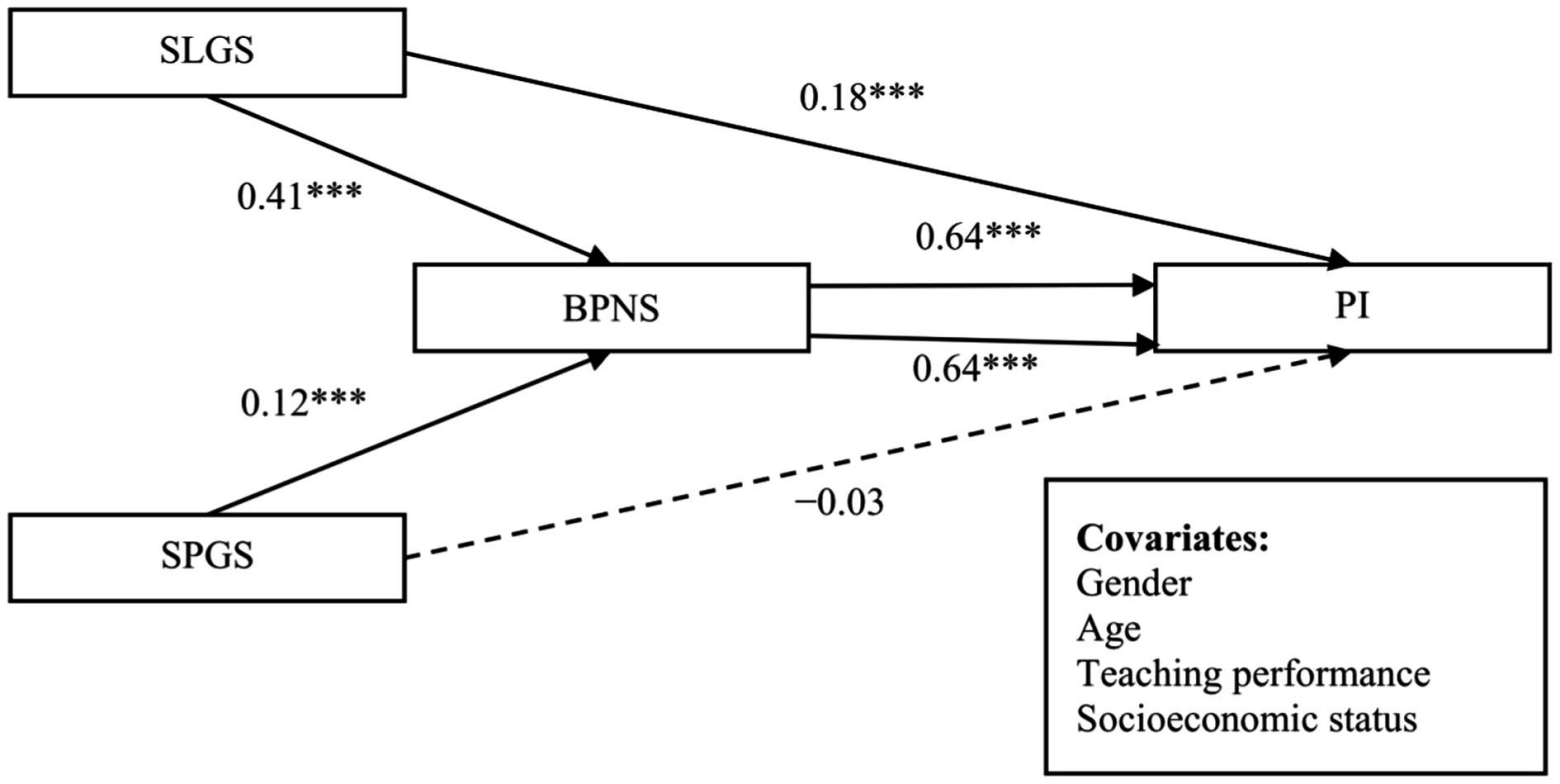
Mediating effects of BPNS. ***p < 0.001. SLGS = school learning goal structure; SPGS = school performance goal structure; BPNS = basic psychological needs satisfaction; PI = professional identity.

We examined the mediating effect of BPNS on the relationship between school learning goal structure and professional identity. As shown in Table 2 and Figure 2, school learning goal structure was positively correlated with BPNS (*β* = 0.41, *SE* = 0.02, *p* < 0.001) and professional identity (*β* = 0.18, *SE* = 0.02, *p* < 0.001). BPNS was positively connected with professional identity (*β* = 0.64, *SE* = 0.02, *p* < 0.001). Further analysis using the bias-corrected bootstrap technique (*n* = 5,000) demonstrated a significant mediating effect of BPNS (*β* = 0.26, *SE* = 0.02, 95% CI [0.23, 0.31]). Therefore, BPNS significantly mediated the relationship between school learning goal structure and professional identity.

**Table 2:** Test of the mediating effect of BPNS on the relationship between school learning goal structure and professional identity.

		*β*	*SE*	*t*	95% CI	*R^2^*	*F*
Equation 1: BPNS	SLGS	0.41	0.02	17.52***	[0.37, 0.46]	0.27	91.60***
	Gender	0.03	0.13	1.19	[−0.10, 0.42]		
	Age	0.16	0.02	4.87***	[0.07, 0.16]		
	TP	0.09	0.02	4.00***	[0.05, 0.14]		
	SES	0.09	0.02	3.84***	[0.04, 0.13]		
	SPGS	0.12	0.02	5.20***	[0.08, 0.17]		
Equation2: PI	SLGS	0.18	0.02	8.91***	[0.14, 0.22]	0.57	281.70***
	BPNS	0.64	0.02	31.85***	[0.60, 0.68]		
	Gender	0.09	0.10	0.83	[−0.12, 0.29]		
	Age	0.04	0.02	2.14*	[0.00, 0.07]		
	TP	0.07	0.02	3.66***	[0.03, 0.10]		
	SES	−0.01	0.02	−0.36	[−0.04, 0.03]		
	SPGS	−0.03	0.02	−1.41	[−0.06, 0.01]		

*p < 0.05, ***p < 0.001. Gender was coded as 0 = man and 1 = woman. TP = teaching performance; SES = socioeconomic status; SLGS = school learning goal structure; SPGS = school performance goal structure; BPNS = basic psychological needs satisfaction; PI = professional identity.

Furthermore, we examined the mediating effect of BPNS on the relationship between school performance goal structure and professional identity. As shown in Table 3 and Figure 2, school performance goal structure was positively correlated with BPNS (*β* = 0.12, *SE* = 0.02, *p* < 0.001); however, it was not significantly connected with professional identity (*β* = −0.03, *SE* = 0.02, *p* > 0.05). BPNS was positively associated with professional identity (*β* = 0.64, *SE* = 0.02, *p* < 0.001). Further analysis using the bias-corrected bootstrap technique (*n* = 5,000) demonstrated a significant mediating effect of BPNS (*β* = 0.08, *SE* = 0.02, 95% CI [0.05, 0.11]). Therefore, BPNS significantly mediated the relationship between school performance goal structure and professional identity.

**Table 3:** Test of the mediating effect of BPNS on the relationship between school performance goal structure and professional identity.

		*β*	*SE*	*t*	95% CI	*R^2^*	*F*
Equation 1: BPNS	SPGS	0.12	0.02	5.20***	[0.08, 0.17]	0.27	91.60***
	Gender	0.16	0.13	1.19	[−0.10, 0.42]		
	Age	0.11	0.02	4.87***	[0.07, 0.16]		
	TP	0.09	0.02	4.00***	[0.05, 0.14]		
	SES	0.09	0.02	3.84***	[0.04, 0.13]		
	SLGS	0.41	0.02	17.52***	[0.37, 0.46]		
Equation2: PI	SPGS	−0.03	0.02	−1.41	[−0.06, 0.01]	0.57	281.70***
	BPNS	0.64	0.02	31.85***	[0.60, 0.68]		
	Gender	0.09	0.10	0.83	[−0.12, 0.29]		
	Age	0.04	0.02	2.14*	[0.00, 0.07]		
	TP	0.07	0.02	3.66***	[0.03, 0.10]		
	SES	−0.01	0.02	−0.36	[−0.04, 0.03]		
	SLGS	0.18	0.02	8.91***	[0.14, 0.22]		

*p < 0.05, ***p < 0.001. Gender was coded as 0 = man and 1 = woman. TP = teaching performance; SES = socioeconomic status; SLGS = school learning goal structure; SPGS = school performance goal structure; BPNS = basic psychological needs satisfaction; PI = professional identity.

### 3.3 Tests of moderating effects of growth mindset

We examined the moderating effect of growth mindsets on the relationship between school learning goal structure and professional identity (Table 4). The results indicated that the interaction effect of school learning goal structure and growth mindset on BPNS was not significant (*β* = −0.02, *t* = 0.03, *p* > 0.05). This result indicated that the moderating effect of a growth mindset on the relationship between school learning goal structure and BPNS was not significant.

**Table 4:** Test of the moderating effect of growth mindset on the relationship between school learning goal structure and professional identity.

		*β*	*SE*	*t*	95% CI	*R^2^*	*F*
Equation 1: BPNS	SLGS	0.40	0.02	16.58***	[0.35, 0.44]	0.29	74.48***
	GM	0.13	0.02	5.35***	[0.08, 0.17]		
	SLGS × GM	−0.02	0.03	−0.61	[−0.07, 0.04]		
	Gender	0.16	0.13	1.24	[−0.10, 0.42]		
	Age	0.12	0.02	5.21***	[0.08, 0.17]		
	TP	0.09	0.02	3.68***	[0.04, 0.13]		
	SES	0.08	0.02	3.46***	[0.03, 0.12]		
	SPGS	0.13	0.02	5.53***	[0.08, 0.17]		
Equation2: PI	SLGS	0.18	0.02	8.91***	[0.14, 0.22]	0.57	281.70***
	BPNS	0.64	0.02	31.85***	[0.60, 0.68]		
	Gender	0.09	0.10	0.83	[−0.12, 0.29]		
	Age	0.04	0.02	2.14*	[0.00, 0.07]		
	TP	0.07	0.02	3.66***	[0.03, 0.10]		
	SES	−0.01	0.02	−0.36	[−0.04, 0.03]		
	SPGS	−0.03	0.02	−1.41	[−0.06, 0.01]		

*p < 0.05, ***p < 0.001. Gender was coded as 0 = man and 1 = woman. TP = teaching performance; SES = socioeconomic status; SLGS = school learning goal structure; SPGS = school performance goal structure; BPNS = basic psychological needs satisfaction; GM = growth mindset; PI = professional identity.

Furthermore, we examined the moderating effect of growth mindsets on the relationship between school performance goal structure and professional identity (Table 5). The results revealed that the interaction effect of school performance goal structure and growth mindset on BPNS was significant (*β* = −0.04, *t* = 0.02, *p* < 0.05). This result indicated that the moderating effect of a growth mindset on the relationship between school performance goal structure and BPNS was significant.

**Table 5:** Test of the moderating effect of growth mindset on the relationship between school performance goal structure and professional identity.

		*β*	*SE*	*t*	95% CI	*R^2^*	*F*
Equation 1: BPNS	SPGS	0.13	0.02	5.74***	[0.09, 0.18]	0.29	74.48***
	GM	0.11	0.02	4.83***	[0.07, 0.16]		
	SPGS × GM	−0.04	0.02	−2.14*	[−0.07, −0.00]		
	Gender	0.17	0.13	1.32	[−0.08, 0.43]		
	Age	0.12	0.02	5.22***	[0.08, 0.17]		
	TP	0.09	0.02	3.77***	[0.04, 0.13]		
	SES	0.08	0.02	3.53***	[0.04, 0.12]		
	SLGS	0.40	0.02	16.70***	[0.35, 0.44]		
Equation2: PI	SPGS	−0.03	0.02	−1.41	[−0.06, 0.01]	0.57	281.70***
	BPNS	0.64	0.02	31.85***	[0.60, 0.68]		
	Gender	0.09	0.10	0.83	[−0.12, 0.29]		
	Age	0.04	0.02	2.14*	[0.00, 0.07]		
	TP	0.07	0.02	3.66***	[0.03, 0.10]		
	SES	−0.01	0.02	−0.36	[−0.04, 0.03]		
	SLGS	0.18	0.02	8.91***	[0.14, 0.22]		

*p < 0.05, ***p < 0.001. Gender was coded as 0 = man and 1 = woman. TP = teaching performance; SES = socioeconomic status; SLGS = school learning goal structure; SPGS = school performance goal structure; BPNS = basic psychological needs satisfaction; GM = growth mindset; PI = professional identity.

**Figure 3:**
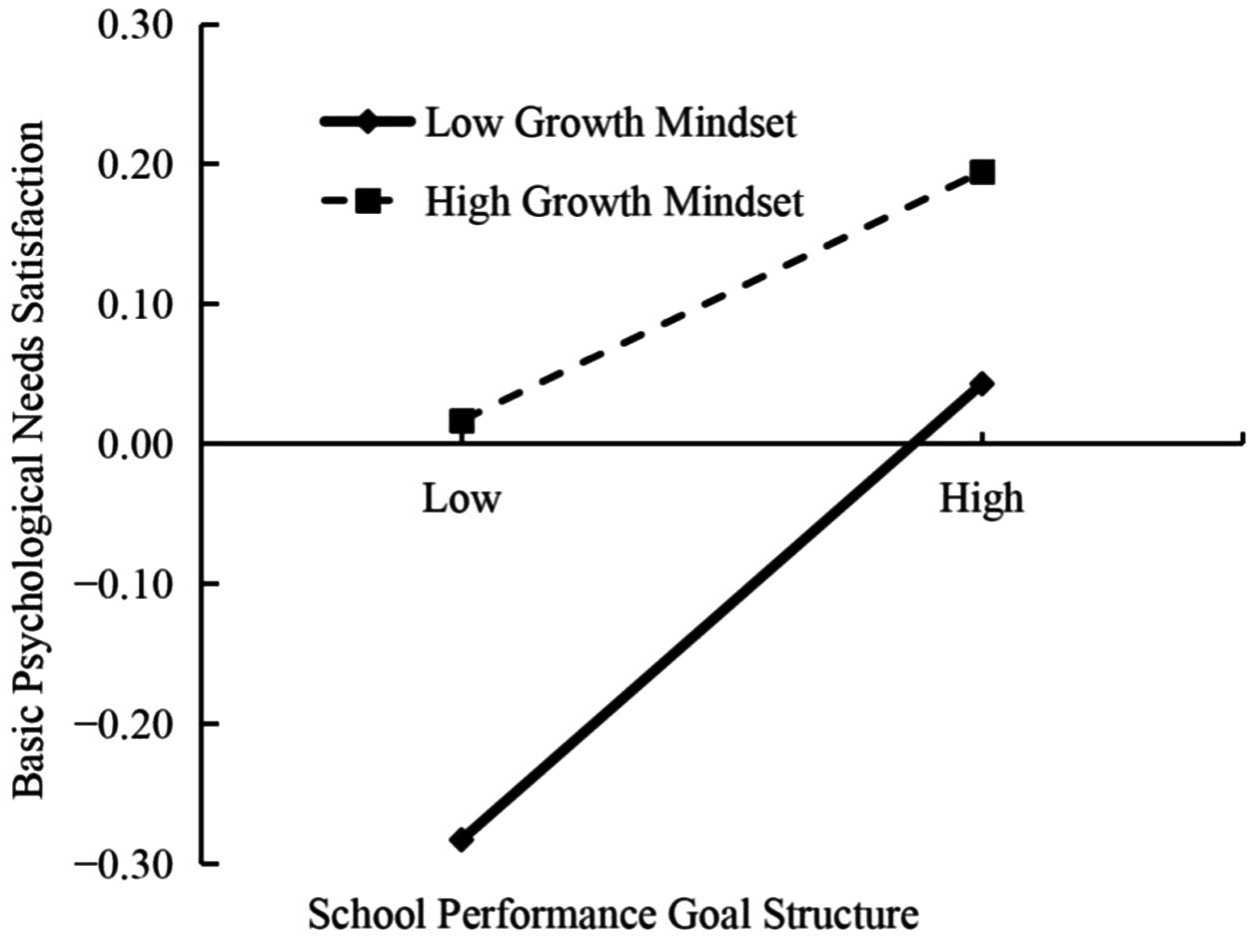
The moderating effect of growth mindset on the relationship between school performance goal structure and basic psychological needs satisfaction.

We performed a simple slope analysis to further investigate the moderating role of growth mindsets in the relationship between school performance goal structure and professional identity. A standard deviation above and below the mean (*M* ± *SD*) was used to calculate the effect value of school performance goal structure on BPNS. As shown in Figure 3, teachers with strong growth mindsets experienced higher BPNS than those with weak growth mindsets, regardless of their school performance goal structure level. Teachers with weak growth mindsets exhibited stronger associations between school performance goal structure and BPNS (*β* = 0.17, *SE* = 0.03, 95% CI [0.11, 0.23], *p* < 0.001) than those with strong growth mindset (*β* = 0.09, *SE* = 0.03, 95% CI [0.04, 0.15], *p* < 0.001). In addition, this study examined whether the mediating effect of school performance goal structure on professional identity via BPNS was conditioned by growth mindsets. The result revealed that the mediating effects of BPNS were stronger among teachers with weak growth mindsets (*β* = 0.11, *SE* = 0.02, 95% CI [0.07, 0.16]) than those with strong growth mindsets (*β* = 0.06, *SE* = 0.02, 95% CI [0.03, 0.09]).

## 4 Discussion

### 4.1 Relationship between perceived school goal structure and professional identity

The findings underscored the intricate relationship between kindergarten teachers’ perceived school goal structures and their professional identity. Specifically, the results indicated that school learning and performance goal structures indirectly affected professional identity through BPNS. However, only the school learning goal structure directly affected professional identity, partially supporting H1. This distinction is crucial, as it highlights the differential impact of school goal structures on teachers’ professional development and wellbeing.

The positive correlation between school learning goal structure and professional identity expands previous research (Skaalvik and Skaalvik, 2023; Sun et al., 2025), suggesting that a mastery-oriented environment emphasizing continuous learning and personal growth fosters strong professional identity among teachers. In such environments, teachers are likely to feel valued for their contribution to students’ holistic development, enhancing their sense of belonging and commitment to their profession. Notably, the absence of a direct effect of the school performance goal structure on professional identity suggests that professional identity stems not only from external evaluations and recognition but also, more importantly, from teachers’ internal experiences and feelings. The school performance goal structure cannot fully capture teachers’ professional identity. Only when it influences teachers’ BPNS and intrinsic motivation can it further affect their professional identity development.

### 4.2 Mediating effect of basic psychological needs satisfaction

BPNS mediated the relationship between the two forms of perceived school goal structures and professional identity. This finding supports H2 and is in accordance with the self-determination theory (Deci and Ryan, 1985). This result extends previous research indicating that BPNS mediates the relationship between the two forms of perceived school goal structures and professional identity. Previous research suggests that a learning environment that focuses on competence (such as a school learning goal structure) provides appropriate support for individuals’ autonomy and competence, thereby facilitating their BPNS (Ameloot et al., 2024). Conversely, school performance goal structure, which overly emphasizes external evaluation and competition, could undermine teachers’ perceived autonomy and competence, and potentially lower their sense of belonging (Ketonen and Nieminen, 2025; Nerstad et al., 2020; Schwarz-Franco and Hadar, 2024). However, in contrast to previous studies, this investigation found that school learning and performance goal structures were positively associated with BPNS. This could be because teachers might perceive the organization’s attention and support as satisfying the three basic psychological needs if the external evaluation within a performance goal structure is constructive.

Another possible explanation is that, within the collectivist cultural context of China, school performance goal structure may carry different meanings than those in individualist Western culture. In collectivist cultures, individual behavior and achievements are often closely linked to the group’s interests. Therefore, Chinese teachers may be more inclined to view external evaluations (e.g., performance appraisals and competition) as important sources of social recognition and collective achievement rather than mere pressure or anxiety (Luo et al., 2020). In China, parents greatly emphasize their children’s education and academic performance in kindergarten. Many children aged 3–6 are enrolled by their parents in full-day early childhood education programs in kindergartens (Gao et al., 2024), such as early English instruction (Shi and Yeung, 2024). Amid this cultural context and societal expectations, teachers who perceive a high-performance school goal structure may view the school’s emphasis on student achievement as an opportunity to gain evaluation and recognition, which can satisfy their basic psychological needs, such as a sense of belonging and competence. This finding suggests that educational administrators should fully consider the influence of cultural background when designing and implementing school goal structure to target the promotion of teachers’ professional development and mental health.

### 4.3 Moderating effect of growth mindset

Consistent with H3, we found that growth mindsets significantly moderated the connection between school performance goal structure and BPNS. Specifically, regardless of the degree to which the school leans toward a performance goal structure, teachers with strong growth mindsets exhibited higher satisfaction with their BPNS than those with weak growth mindsets. This finding aligns with past research indicating that the school working environment can interact with a growth mindset to jointly influence teachers’ professional development (Zhang and He, 2024). Mindset influences the cognitive processing of information, thereby affecting judgments of personal self-efficacy (Bardach et al., 2024). Teachers with strong growth mindsets typically believe in the malleable nature of teaching (for instance, the ability to complete teaching tasks flexibly and professionally) and think they can overcome obstacles by taking action (Yeager and Dweck, 2012). Therefore, when facing job demands imposed by the school performance goal structure, they are motivated to exert effort to adapt and adjust their teaching strategies and methods (Lin et al., 2022). In this process of continuous self-transcendence, teachers also experience satisfaction with basic psychological needs such as competence. This result supports Dweck’s social-cognitive framework (Dweck and Leggett, 1988) and the job demands-resources model (Bakker and Demerouti, 2007).

Notably, the effect of the school performance goal structure on BPNS was weaker among teachers with strong growth mindsets than among those with weak growth mindsets. In other words, as schools increasingly leaned toward adopting performance goal structure, the positive impact of a growth mindset paradoxically diminished. This indicates that the school performance goal structure undermines the positive effect of a growth mindset. Mindsets guide individuals toward different types of achievement goals (Dweck and Yeager, 2019). Teachers with strong growth mindsets believe that effort can alter ability and tend to pursue mastery goals (Bardach et al., 2024). Consequently, these mindsets may conflict with the school performance goal structure (Skaalvik and Skaalvik, 2023). In this context, teachers with a strong growth mindset may need to expend considerable psychological effort to adapt to the school performance goal structure, which diminishes the positive impact of a growth mindset on teachers’ BPNS.

However, contrary to H3, the results showed that growth mindsets did not significantly moderate the relationship between school learning goal structure and BPNS among kindergarten teachers. This finding is inconsistent with past research that has identified a positive moderating role of growth mindset (Zhang and He, 2025) but can be explained by a recent study (Lee et al., 2023). This study argues that teachers with a stronger growth mindset have a positive view of the program when perceived resources and support for implementing a positive education program are insufficient. In contrast, their growth mindsets are unrelated to their views of the program when perceived resources and support are sufficient. Similarly, schools with a performance goal structure tend to lead to social comparison and competition between teachers and students and provide insufficient positive resources (Anderman et al., 2024; Skaalvik and Skaalvik, 2023). Therefore, a growth mindset has a significant moderating effect in this context. However, schools with a learning goal structure also show a high level of concern for teachers’ personal growth, which is conducive to meeting teachers’ basic psychological needs and enhancing teachers’ wellbeing (Wang et al., 2014). Therefore, in such a school culture, whether teachers have a growth mindset is less relevant.

### 4.4 Implications and limitations

This study offers a novel perspective on the factors influencing kindergarten teachers’ professional identity. Our findings hold significant theoretical implications for future studies and provide meaningful insights for practice and policy. First, the results demonstrated the critical role of school goal structures and indicated that only school learning goal structure effectively enhanced kindergarten teachers’ professional identity. This finding underscores that schools can shift the focus of education toward mastery of content rather than the pursuit of competition and good performance. Communicating these educational goal and values to teachers can strengthen their professional identity. Second, our findings revealed the crucial mediating effect of BPNS on the relationship between perceived school goal structures and teachers’ professional identity. This result indicates that BPNS is a core driver of teachers’ professional identity. Therefore, kindergarten leaders should encourage teachers to have greater voice and agency in decision-making processes and to exercise more autonomy in their professional practices. Finally, the results highlight the significant moderating role of growth mindsets in the relationship between school goal structures and professional identity among kindergarten teachers. Therefore, supporting teachers in developing teaching practices that align with a growth mindset is crucial. Kindergarten leaders should find ways to educate teachers on adopting a growth mindset in learning and implementing more growth-oriented teaching strategies. For example, they can cultivate teachers’ mastery-oriented teaching, normalize challenges and mistakes in learning, and focus on feedback and assessment that emphasize process and effort rather than performance and personal ability (Laine and Tirri, 2023).

This study has some limitations. First, the cross-sectional design prevented the identification of causal relationships. Future research should employ longitudinal designs to validate these findings further. Second, the data collection in this study relied on teachers’ self-reports. This single-source method may have introduced a subjective bias, thereby affecting the objectivity of the results. To minimize the potential social desirability effects, future studies should explore diverse information sources, such as incorporating reports from parents and colleagues, to form a comprehensive and objective dataset. Third, the population of kindergarten teachers in China is predominantly female, and our sample similarly exhibits this gender imbalance, which aligns with prior research (Ren et al., 2024). Although we controlled for gender in our statistical analyses to mitigate its potential influence on the results, this influence cannot be eliminated. Future research should consider separately comparing the two distinct groups of female and male teachers to further explore gender differences in the impact of perceived school goal structures on teachers’ professional identity. Finally, as this study focused on kindergarten teachers in China, our conclusions may be limited to China’s specific educational environment and social context. To test the generalizability of the findings, future research should expand the sample size and compare the impact mechanisms of perceived school goal structures on professional identity across various cultural backgrounds.

## 5 Conclusion

This study examined the differences in the underlying mechanisms of the impact of two types of perceived school goal structures on professional identity among kindergarten teachers in China. The results suggest that kindergartens should actively construct a learning goal structure to enhance teachers’ professional identity. Additionally, both types of perceived school goal structures enhance BPNS, which in turn promotes the development of professional identity among kindergarten teachers. Notably, the growth mindset exhibited differential moderating effects on these relationships, specifically moderating the relationship between school performance goal structure and BPNS. This study lays a solid theoretical foundation for developing effective interventions to enhance teachers’ professional quality and promote educational development. However, the generalizability of the research findings may be affected by the self-report method and the limitations of the sample. Hence, future research should employ more diverse data collection methods to further explore the specific impact of perceived school goal structures on kindergarten teachers’ professional identity across different cultural contexts, thereby deepening the understanding of this field.

## Data Availability

The raw data supporting the conclusions of this article will be made available by the authors, without undue reservation.

## Footnotes

^1^ www.wjx.cn
